# DNA Replication Licensing Factors: Novel Targets for Cancer Therapy via Inhibiting the Stemness of Cancer Cells

**DOI:** 10.7150/ijbs.67529

**Published:** 2022-01-01

**Authors:** Shaoran Song, Yaochun Wang, Peijun Liu

**Affiliations:** 1 Center for Translational Medicine, the First Affiliated Hospital of Xi'an Jiaotong University, Xi'an, China; 2 The Key Laboratory for Tumor Precision Medicine of Shaanxi Province, The First Affiliated Hospital, Xi'an Jiaotong University, Xi'an, Shaanxi 710061, China

**Keywords:** replication initiation factors, cancer stem cell, stemness, drug resistance

## Abstract

The replication licensing factors strictly regulate the DNA replication origin licensing process to guarantee the stability of the genome. Numerous experimental studies have recently demonstrated that the replication licensing factors as oncogenes are essential for the occurrence and development of cancers. Drug resistance, being one of the main characteristics of cancer stem cells, can cause a high recurrence rate and a low survival rate in patients with different cancers. However, the function of the replication licensing factors in cancer stemness remains unclear. The following article highlights the most recent research on DNA replication origin licensing factors in cancer and their function in anti-cancer drug resistance. Moreover, this article proposes a new perspective that replication licensing factors as chemotherapy shield affect anti-cancer drug resistance by promoting the stemness of cancer cells.

## Introduction

Cancer is anticipated to be the leading cause of mortality worldwide in the twenty-first century, posing the most significant impediment to extending life expectancy. According to estimates from the GLOBOCAN in 2020, there were 19.3 million new cancer cases and 9.9 million cancer deaths worldwide 1. Although traditional treatment methods such as anti-cancer drugs, surgical resection, combined chemotherapy, and radiotherapy have achieved significant results, increasing numbers of patients inevitably face higher tumor recurrence rates and metastasis. The aggressive characteristics of cancer are partially due to cancer stem cells (CSCs) being resistant to conventional anti-cancer treatments.

**Figure 1:**
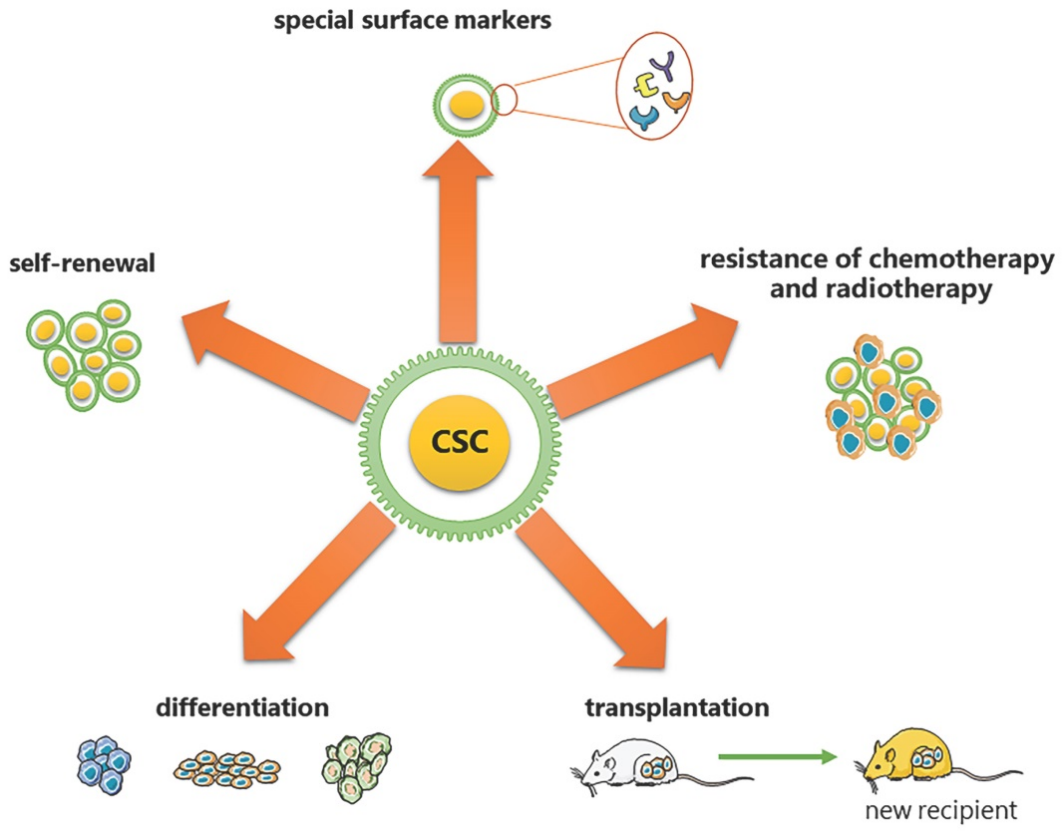
Five most characteristic properties of CSCs. (1) self-renewal: tumor-sphere formation in vitro/ tumorigenic ability in vivo; (2) the ability of differentiation; (3) the ability of transplantation; (4) resistance of conventional chemotherapy and radiotherapy; (5) unique surface markers.

CSCs are considered to have the exclusive ability to grow malignant cell populations indefinitely, which is recognized as the primary cause of cancer recurrence and metastasis 2-5. Pierce and colleagues described CSCs for the first time as malignant cells with a high proliferative ability and a limited capacity for differentiation under normal homeostatic conditions 6. Nowadays, a substantial body of research has established that CSCs are critical in a variety of tumor types, including leukemia 7, prostate cancer 8, breast cancer 9, and colorectal cancer 10. Based on the analysis from previous research, CSCs have been proven to have the following five most characteristic properties (as shown in Figure 1): (1) self-renewal: tumor-sphere formation in vitro and tumorigenic ability in vivo 11, 12; (2) the ability of differentiation; (3) the ability of transplantation 13; (4) resistance of conventional chemotherapy and radiotherapy 14; and (5) unique surface markers 15. Additionally, CSCs were verified to be dormant 15. Moreover, identifying and isolating CSCs from solid tumors based on their characteristics has been widely employed to develop more effective strategies for cancer eradication. However, the hierarchical structure of CSCs is shown to be more malleable than previously believed, complicating their complete elimination 2.

DNA replication in eukaryotic cells requires a precise synthesis process. However, with long-term amplification, DNA replication errors continue to accumulate, leading to genome instability, which is one of the hallmarks of cancer 16-18. Furthermore, increased genomic instability accelerates clonal evolution, resulting in more aggressive clones and stubborn drug resistance 19. As a result, controlling DNA replication's origin is a critical mechanism for cancer elimination 20. Moreover, the determination of DNA replication origins in eukaryotes involves two subsequent steps: (1) “licensing”: identification of the pre-RC site; (2) “firing”: activation of DNA synthesis 21. "Licensing" is a critical step in enforcing spatial and temporal constraints on DNA replication. Factors that participate in DNA replication origin licensing include the origin recognition complex (ORC, comprising the six subunits ORC1-6), cell division cycle 6 (CDC6), CDC10‑dependent transcript 1, (also known as DNA replication factor CDT1), and the mini-chromosome maintenance (MCM) helicase complex. These licensing factors have been identified as oncogenes, making them attractive potential therapeutic targets 22. Furthermore, there is mounting evidence that dysregulation of DNA replication licensing factors may influence cancer occurrence and progression via regulating CSCs stemness maintenance 23, 24.

This article highlights the current DNA replication licensing factors and their role in drug resistance in normal stem cells and CSCs, significantly increasing the replication licensing factors' potential as novel therapeutic targets. However, more research is urgently needed to elucidate the regulatory mechanisms of DNA replication licensing factors and their related pathways for the differentiation, self-renewal, and differentiation of cancer stem cells, which may eventually strengthen our ability to promote cancer treatment and prevention technologies through the elimination of cancer stem cells.

## Brief of DNA Replication Origin Licensing

**Figure 2:**
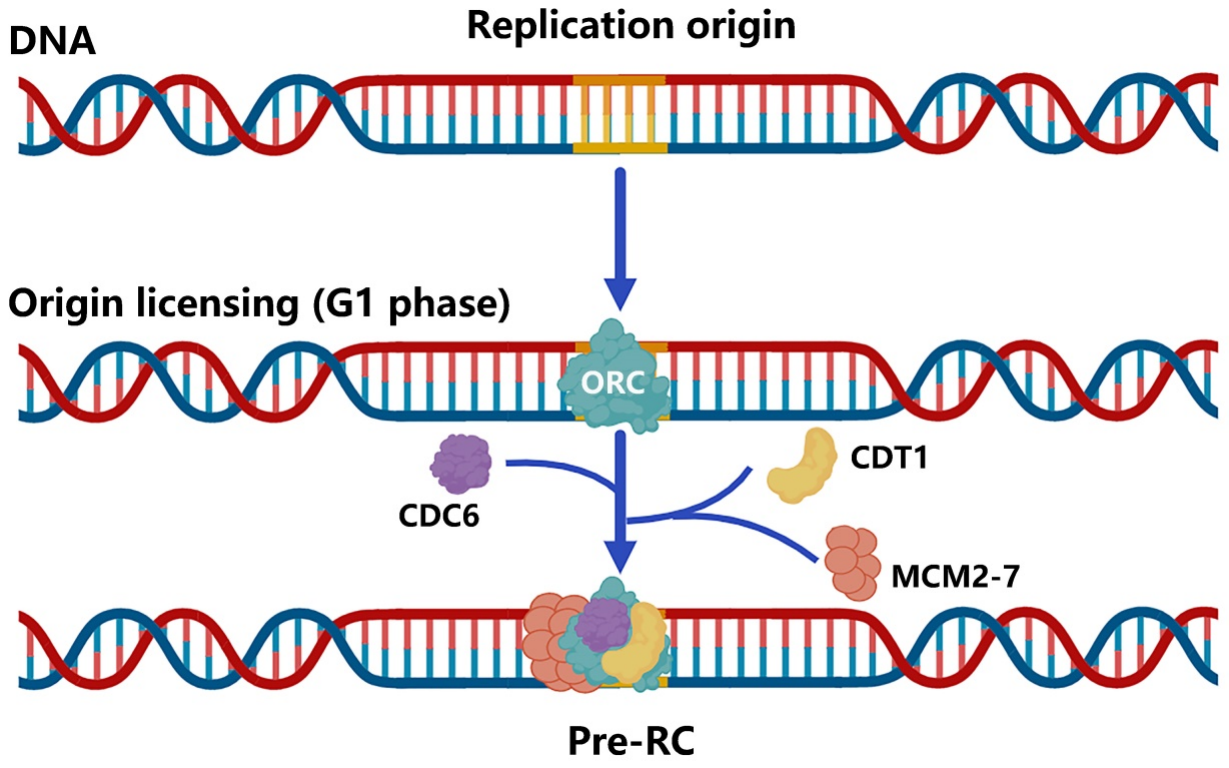
Brief of DNA replication origin licensing. Sequential loading of replication licensing factors on all potential origins in the genome makes replication initiation restricted to the G1 phase. Firstly, the origin recognition complex (ORC, comprising the six subunits ORC1-6) with ATPase activity is recruited to replication origins, followed by cell division cycle 6 (CDC6) and CDC10-dependent transcript 1 (CDT1) binding to the ORC, and finally the mini-chromosome maintenance helicase complex (MCM2-7) loading into the complex to form Pre-RC.

DNA replication origin licensing requires a series of different proteins to act in turn. To begin with, the evolutionarily conservative ORC features a gap that permits DNA to enter through the pentameric ORC ring's central channel and interact with it via ATP 25, 26. These binding sites, known as replication origins, are essential for loading replicative helicases 27-29. Using cryo-EM, Li et al. revealed that subunits of ORC could cooperate with the initiator specific motif (ISM) and β-hairpins to bend DNA, which is essential for the loading of MCM2-7 30. Second, CDC6, another AAA+ ATPase, loads to the replication origin and seals the previous gap on ORC, trapping DNA in the ORC⸱CDC6 toroid 25, 26. However, the mechanism by which CDC6 regulates the loading of MCM2-7 through the change of the ATP hydrolysis cycle remains unclear. Simultaneously, MCM2-7 and CDT1 form a stable OCCM complex to ensure that the opening of the hexamer can smoothly pass through the DNA that has already been loaded with ORC and CDC6 31. The research of Yuan et al. discovered that both CDC6 and ORC2 interact directly with the MCM3 WH domain 32. Thus, these factors collectively form the pre-initiation complex (pre-RC), which "licenses" the origins to replicate DNA and progress to the next phase of the cell cycle 21 (Figure 2).

## Replication Origin Licensing and Cancers

To ensure the high accuracy of replication, strictly control only one replication origin licensing per cell cycle. Once inappropriate replication origin licensing occurs in the same cell cycle, it amplifies the nuclear genome, a process known as re-replication. Mutations such as overexpression of CDT1/ CDC6 or depletion of geminin have been reported to lead to re-replication rapidly 33, 34. Besides that, Re-replication is usually accompanied by the appearance of DNA damage, genomic stress, or instability, which is related to cell cycle arrest, senescence, and apoptosis. Thus, replication licensing is highly associated with multiple clinical pathogenesis and tumorigenesis.

### Replication origin licensing factors and cell cycle progression and proliferation

The rapid and uncontrolled proliferation caused by multiple genetic mutations is necessary for carcinogenesis 16, 17, 35. Uncontrolled proliferation and/ or enhanced genomic instability make most tumor cells suffer from high replication stress. Consistent with this, it has been reported that replication licensing factors are overexpressed in various cancer cell lines and function as oncogenes 36-38. Since only licensed DNA is allowed to enter the S phase from the G1 phase. The destruction of replication licensing can lead to G1-S phase arrest, inhibiting cell proliferation. Previous studies have demonstrated decreased ORC6 expression induced by the siRNA knock-down approach triggered cell cycle arrest in the G1 phase. This cell cycle regulation is associated with wild-type p53 37. Moreover, the normal loading of MCM proteins onto chromatin during the G1 phase was impeded after the deletion of either CDC6 or CDT1, thereby stalling the cell cycle progression 39. In vitro studies on mouse embryonic fibroblasts and fetal and adult diploid tissues revealed that global or tissue-specific ORC1 deficiency impairs DNA replication, cell lineage expansion, and organ development 40.

### Replication origin licensing factors and cell apoptosis and survival

Reduced expression of licensing factors leads to more apoptosis in different cancers. In cell lines of gastric cancer, osteosarcoma, and cervical cancer, down-regulation of CDC6 promotes cell apoptosis, which negatively impacts growth in vivo and in vitro 40-42. Moreover, partial overexpression of MCMs can enhance cell proliferation significantly and suppress apoptosis, whereas knockdown of MCMs reverses these effects 43-45. Notably, subcellular localization of replication licensing factors was found to correlate significantly with cell apoptosis in cancer cells. The perinuclear accumulation of ORC1 caused by the absence of normal ORC1 modifications such as monoubiquitylation and hyperphosphorylation was demonstrated to induce cell apoptosis.

Similarly, ORC2 could restore the uniform nuclear localization of ORC1 and prevent the induction of apoptosis 41. Furthermore, since increased cytoplasmic MCM2 promotes cell apoptosis, tumor samples with cytoplasmic MCM2 demonstrated better prognoses 42. Hence, targeting replication licensing factors through epigenetic modification or subcellular localization may be an appealing strategy for inducing cancer cell death and improving survival.

### Replication origin licensing factors and metastasis

Metastasis consists of two major steps: dissemination and colonization. Numerous investigations have implicated replication licensing factors as the key molecules regulating almost all the steps of metastasis by targeting key genes. By suppressing the ERK/JNK signaling pathway, decreased ORC1 inhibited cancer cell invasion and migration 43. Trans-well migration assays demonstrated that silencing CDC6 dramatically reduced the ability of UMUC3 and T24 cells to migrate 39. In HCT-116 cells, CDC6 overexpression was associated with the reduction of E-cadherin, indicating CDC6 may have an important role in the metastasis of HPV-associated cancers 44. A similar regulating effect was identified in Sideridou's research 45. Consequently, replication licensing factors play an important role in cancer metastasis.

## Replication Origin Licensing and Drug-Resistance

### ORC and drug-resistance

The ORC complex serves as a platform for initial assembly during the DNA replication process. Various studies have been conducted to determine its role in the development and treatment of cancers and how its aberrant expression affects the susceptibility to traditional cancer therapy. For example, it has been observed that in colon cancer cells HCT-116 (wt-p53) with lower expression of ORC6 were more sensitive to 5-fluorouracil (5-FU) and cisplatin treatment than the control group, as a result of p53 phosphorylation regulation 37. In response to gemcitabine treatment, Plk1-mediated phosphorylation of ORC1/2 at the start of replication was elevated, and replication initiation was increased, leading to resistance to chemotherapeutic agents 46. The resistance of gemcitabine may also involve a p53-dependent manner, but the specific molecular mechanism needs further study. Another study suggested that siORC1 enhanced the sensitivity of U2OS cells to hydroxyurea (HU), although the removal of other origin licensing factors, such as ORC6 or CDC6, did not. Nevertheless, in non-tumor cells, the enhanced sensitivity to HU was not detected 47. Compared with non-cancer cells, tumor cells may depend on the origin licensing capacity due to their higher oxidative and replication stress. Consequently, targeting origin licensing factors can make cancer cells more sensitive to chemotherapy drugs.

### MCM2-7 and drug-resistance

The MCM2-7 complex, as the core of the replication initiation permission complex, is also involved in the formation of DNA helicase, which is responsible for the melting and unwinding of the double helix during DNA synthesis 21, 48, 49. Recently, genomic analyses also identified MCMs as gene candidates for acquired drug resistance in several types of cancer 50, 51. MCM2 has been shown to be strongly related to the Vemurafenib resistance induced by up-regulated expression of CDC7 52. Due to a p53-dependent apoptotic response, MCM2 deletion can also increase the sensitivity of ovarian cancer cells to carboplatin 53. Additionally, down-regulation of MUS81 promotes apoptosis by inducing S-phase arrest and activation of MCM2, thereby increasing the sensitivity of EOC cells to Olaparib 54. Microarray analysis revealed that the transcriptional expression of MCM2 in the cisplatin-resistant ovarian carcinoma cell was twice that of the non-drug-resistant group 55. However, the specific mechanism remains to be explored. Research by Mitali Das and colleagues indicated that excessive amounts of MCMs could be used as a backup for replication stress in cervical cancer cells; moreover, its regulatory mechanism of sensitivity to cisplatin depends on the HPV status of the cells 56. Furthermore, recent work by Wang et al. indicated that the EGFR pathway can regulate the interaction of MCM8 and other DNA replication licensing factors, hence preserving the clonogenic and tumorigenic potential of GSCs 57.

Furthermore, lentivirus-mediated MCM7 silencing could significantly sensitize chronic lymphocytic leukemia cells to fludarabine 58. It has also been demonstrated that MCM7 plays an important role in cell cycle arrest, apoptosis, and cell death caused by BVP-induced DNA damage 59. Furthermore, the underlying mechanism involves regulating retinoblastoma protein (Rb) and the expression of checkpoint control proteins 59. Interestingly, statin drugs could reduce the expression of MCM7 and RB via activating ER and autophagy signaling pathways, which induced the growth-inhibitory effects in TamR cells 60, 61. Therefore, these findings suggest that MCMs might be a potential target for drug-resistant cancer cells.

### CDC6 and drug-resistance

Several types of research have revealed that despite its function during DNA replication licensing, CDC6 also regulates mitosis exit from yeast cells to human cells by interacting with Cdk1 62-64. In addition, CDC6 was found to be highly expressed in a variety of cancer cells, including drug-resistant cancer cells 39, 65. Recent studies have elucidated that CDC6 could promote mitotic slippage by inhibiting CDK1; thus, cancer cells avoid apoptosis and exhibit PTX resistance 66. CDC6 was predicted to play a pivotal role in ovarian cancer treated with decitabine 67. Interestingly, both bladder cancer cells and CDDP-resistant bladder cancer cells became more sensitive to CDDP when CDC6 was down-regulated 63. Because the CDDP-induced S phase arrest was abolished under the deletion of CDC6, which led to aberrant mitosis by inactivating the ATR-Chk1-Cdc25C pathway 39. CDC6 and Human antigen R (HuR) were found to be positively correlated with malignant behaviors and oxaliplatin (L-OHP) resistance 68. Mechanistically, it has been proved that HuR can bind to CDC6 3'-UTR, thus regulating the sensitivity of CRC cells to L-OHP 68. Therefore, CDC6 may be a novel molecular target to overcome drug resistance.

### CDT1 and drug-resistance

According to Stathopoulou and colleagues, anticancer chemotherapeutic agents can degrade CDT1 in various ways 69. However, only a few researchers have mentioned a relationship between CDT1 and anti-cancer drug resistance 70, 71. Intriguingly, stability or activation of CDT1 or acute depletion of CDT2 could result in re-replication, which radio-sensitizes head and neck squamous cell carcinoma (HNSCC) cells 72. Numerous studies published in the last few years have clarified CDT1's prognostic function in various types of cancer. However, further investigation requires further investigation to determine whether it has the same effect on anti-cancer treatment resistance as other replication licensing factors (Table 1).

## Replication Origin Licensing Factors and Stemness

### Replication origin licensing factors in normal stem cells

Stemness is the ability of a cell to remain undifferentiated (self-renewal) while having the ability to differentiate into any other cell type (potential) 73. Stemness exits in both normal stem cells and CSCs. According to retrospective investigations, replication licensing factors are required to maintain stem cell pluripotency. MCMs loading is restricted to occurring in the G1 phase to prevent re-replication. Initially, partial depletion of MCMs proteins was confirmed to result in cancer and stem cell deficiencies in vitro and in vivo 74, 75. Although less MCMs loading was sufficient for normal proliferation, cells with excess MCMs loading were more resistant to DNA damage and replication stress 69, 76. Further research has reported that rapid MCMs loading as an inherent feature of stem cells helps maintain pluripotency and slows differentiation 23. Moreover, CDT1 and CDC6 were expressed extensively in pluripotent stem cells, similar to findings from mouse embryonic stem cell research 77.

### Replication origin licensing factors in CSCs

**Figure 3:**
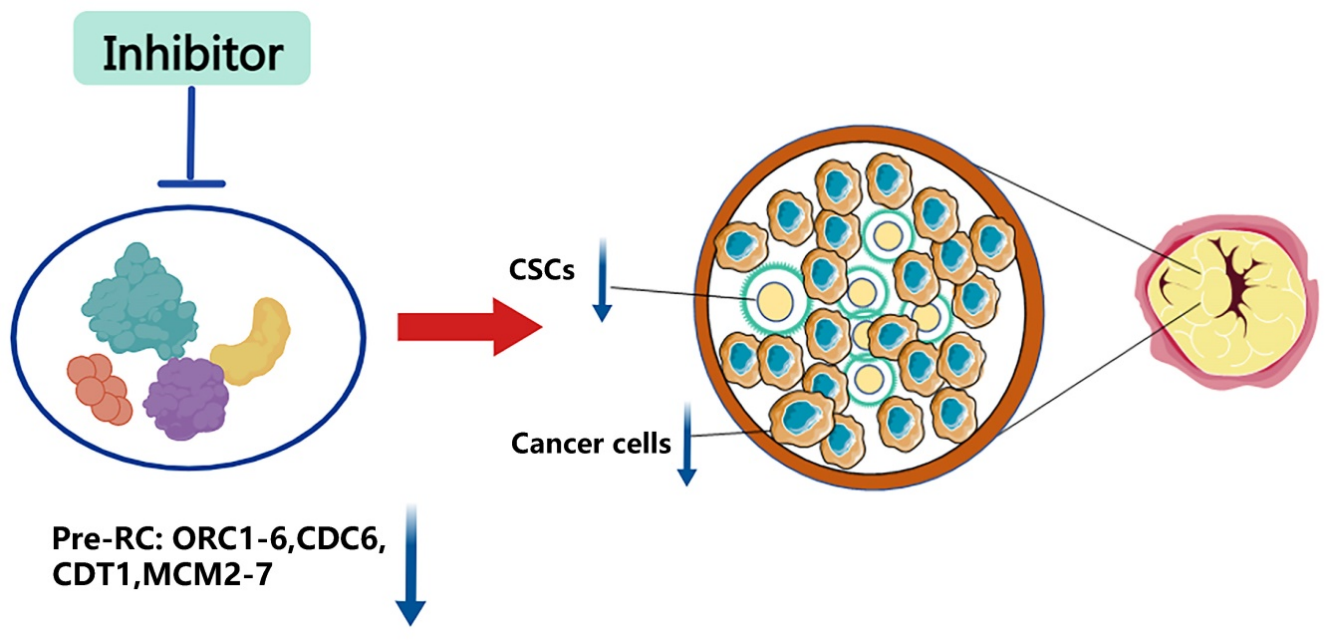
Inhibition of replication origin licensing factors targeting both cancer cells and CSCs. Since replication licensing factor expression is higher in cancer cells, especially in cancer stem cells, than in normal cells, inhibitors that selectively target replication initiation factors could eradicate both cancer cells and cancer stem cells.

Since normal stem cells and CSCs share many characteristics, under certain conditions or stimulation, normal stem cells can be transformed into CSCs. Additionally, cancers will arise when the pluripotent stem cells of epigenetic organisms are inoculated into heterozygous sites in vivo 78-80. Thus, we hypothesize that replication licensing factors could maintain the aggressive characteristics of CSCs, such as rapid proliferation, metastasis, and drug resistance, which were observed in normal stem cells. Numerous experimental studies have also reinforced this view. Mechanically, genes that contribute to stemness regulation, such as p53, c-MYC, and RB, are also proven to exist in the replication licensing pathway regulating cancer stemness 47, 60, 61, 75, 81. As a consequence, replication licensing factors could positively shield cancer cells with stemness against chemotherapeutic treatment. The expression level of replication initiation factors in cancer cells is significantly higher than that of normal cells, and tumor stem cells are even higher than the former. To this end, we propose using inhibitors that specifically target replication initiation factors to destroy tumors. It can extirpate not only normal tumor cells but also CSCs (Figure 3). As a result, further research on selective inhibitors of replication initiation factors is urgently needed in the future.

### Possible mechanisms of tumor recurrence due to replication licensing factors

**Figure 4:**
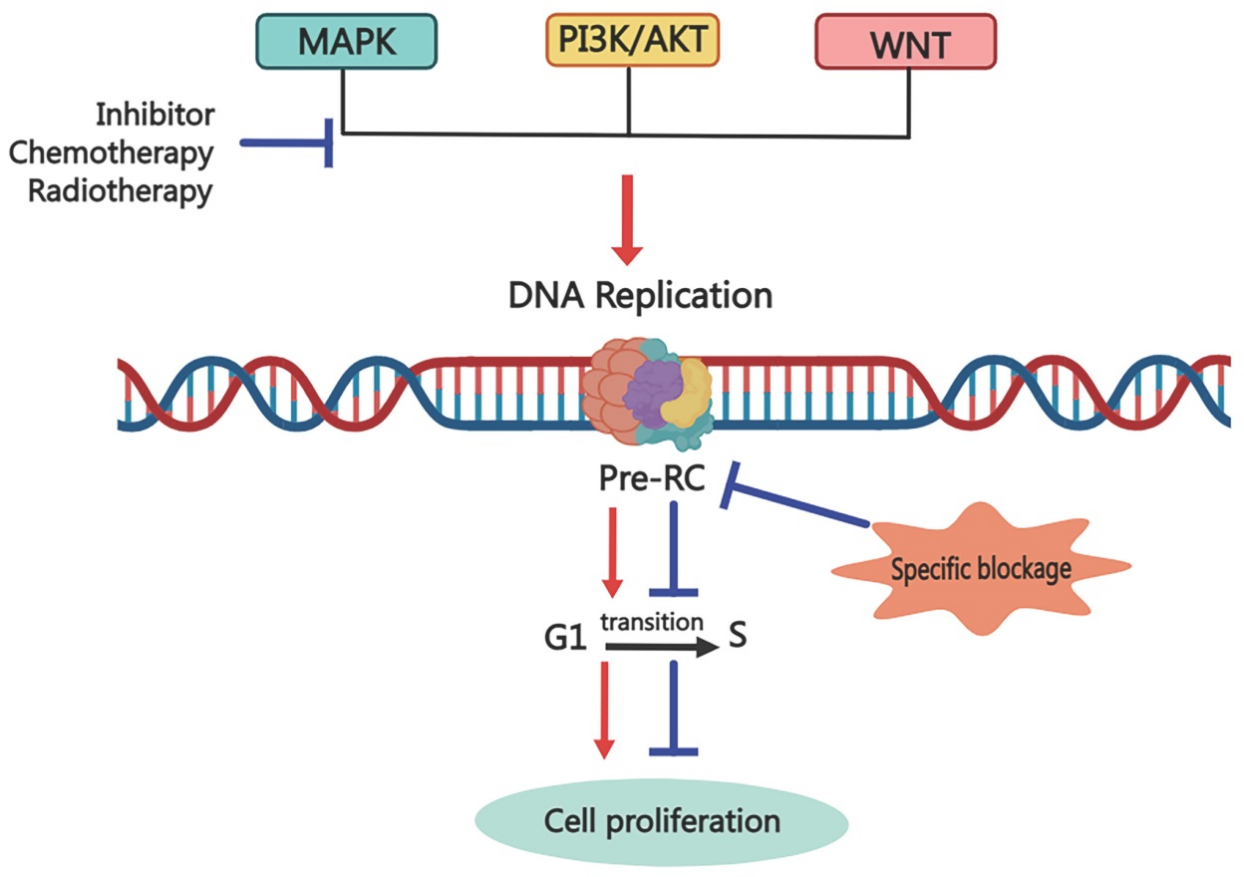
Possible mechanism for targeting Pre-RC to eliminate cancer cells via inhibiting the cancer stemness. Common anti-cancer treatments block one signal transduction pathway to inhibit downstream biological events, including cell proliferation, invasion, and migration. However, cunning tumor cells can trigger cancer recurrence and metastasis by enhancing the transduction of other signaling pathways. Targeting the replication licensing process specifically, which is the foundation for all biological functions, could eradicate cancer thoroughly.

Although there is a relatively complete system for cancer treatment, traditional treatments such as surgery, adjuvant radiotherapy, and chemotherapy can no longer cope with the rapidly increasing rate of recurrence and metastasis. And molecular targeted therapy emerges in time. It is a revolutionary way to selectively target specific molecules with drugs or other substances to prevent cancer cells from proliferation and spread. Although these selective molecular therapy drugs have achieved good results, we have found that patients will still relapse and metastasize after a period of targeted drug treatment. So, what is the root of cancer recurrence and metastasis? Here, we give a possible mechanism for it, as shown in Figure 4. When molecular target therapy agents work on one signal transduction pathway, they block the downstream cell events, including cell proliferation, invasion and metastasis, cell cycle regulation, angiogenesis, and so on. However, after a while, the cunning tumor cells choose to increase the signal transduction of other pathways to promote the downstream cell events, leading to cancer relapse and metastasis. But all the cell events are based on the DNA replication process, and replication licensing is the key step of the DNA replication process. Thus, the destruction of replication licensing factors, the cancer shield, may be a fundamental treatment to eliminate tumors.

## Conclusion and Future Perspectives

This review briefly reviewed the licensing process during replication initiation and the function of replication licensing factors in tumorigenesis and development. The current information on replication licensing factors on the regulation of drug resistance in various cancer cells was given in a summary. By retrospecting the role of the replication licensing factors in stem cells, cancer cells, and cancer stem cells, we found that highly expressed replication licensing factors maintain the aggressive characteristics of CSCs, thus making them stronger than non-stem cancer cells in response to DNA damage and replication stress caused by chemotherapeutic agents. Therefore, we postulated that the replication licensing factors, as a shield for cancer, could maintain the stemness of CSCs and lead to cancer recurrence and metastasis. With the continuous deepening of the understanding and research of cancer stemness, new anti-cancer methods targeting cancer stem cells are needed to reduce the possibility of tumor recurrence. Therefore, understanding the mechanism of the replication licensing factors maintaining stemness and regulating drug resistance can significantly benefit us in cancer research and treatment.

## Figures and Tables

**Table 1:** Replication licensing factors and drug-resistance.

DNA replication licensing proteins	Anti-cancer agent	Cancer cell line	reference
ORC1-6	5-Fluorouracil, cisplatin	HCT-116	37
	gemcitabine	Panc-1, BxPC-3	46
	Hydroxyurea, Hydrogen peroxide	U20S, HeLa, MDA-MB-231	47
CDC6	decitabine	ovarian cancer cell	67
	paclitaxel	MDA-MB-231, HepG2, BGC823, SGC7901	65, 66, 82
	cisplatin	UMUC3	39
	oxaliplatin	HT-29, HCT116	68
MCM2	Vemurafenib	A375, WM983B	52
	carboplatin	A2780	53
	Olaparib	A2780, SKOV3	54
	cisplatin	PE01, PE01CDDP	55
	Trichostatin A	HCT116	71
	Geinstein	LNCaP, PC3	70
MCM4	cisplatin	SiHa, ME180, CaSki, and C-33A,	56
MCM5	camptothecins	MMRU	50
	doxorubicin	AML-2	51
MCM6	pemetrexed	NSCLC cell	83
MCM7	fludarabine	MEC-1, EHEB	58
	cisplatin	T24, T24R2	84
	vinblastine (VBL)	KB-3-1, KB-v1	85
	Oxaliplatin, Etoposide	SW480	86
	Gemcitabine, 5-Fluorouracil	Panc1, Colo-357	86
	Breviscapine	LNCap, PC3, C4-2B	59
	tamoxifen	MCF7 TamR, T47D TamR	61
CDT1	Trichostatin A	HCT116	71
	Geinstein	LNCaP, PC3	70

## Abbreviations

CSCs: cancer stem cells
ORC: origin recognition complex
CDC6: cell division cycle 6
CDT1: CDC10‑dependent transcript 1
MCM: mini-chromosome maintenance helicase complex
pre-RC: pre-initiation complex
HU: hydroxyurea
5-FU: 5-fluorouracil
Rb: retinoblastoma protein
HNSCC: head and neck squamous cell carcinoma
